# Burden, reward, and coping of adult offspring of patients with depression and bipolar disorder

**DOI:** 10.1186/s40345-015-0021-5

**Published:** 2015-01-31

**Authors:** Rita Bauer, Hermann Spiessl, Marina J Helmbrecht

**Affiliations:** Department of Psychiatry and Psychotherapy, University Hospital Carl Gustav Carus, Technical University Dresden, Fetscherstr. 74, 01307 Dresden, Germany; Department of Psychiatry and Psychotherapy, University of Regensburg, Universitätsstraße 84, 93053 Regensburg, Germany; State Hospital for Psychiatry, Psychotherapy and Psychosomatics, Prof.-Buchner-Strasse 22, 84034 Landshut, Germany; Department of Psychiatry and Psychotherapy II, BKH Günzburg, Ulm University, Ludwig-Heilmeyer-Str. 2, 89312 Günzburg, Germany

**Keywords:** Bipolar disorder, Depression, Caregiver, Burden, Reward of caregiving

## Abstract

**Background:**

In previous years, research has focused on the situation of psychiatric patients' minor children. The aims of this qualitative study were to describe the experience of adult children of depressed and bipolar patients, including positive and negative factors as well as coping mechanisms, and to investigate possible predictors of burden in order to identify children in need of professional support.

**Methods:**

A total of 30 adult children were interviewed using a semi-structured interview. In addition, all children completed the Freiburg Questionnaire of Coping with Disease (*Freiburger Fragebogen zur Krankheitsverarbeitung, FKV*). Regression analysis indicated the most relevant predictors of burden.

**Results:**

All (100%) of the children reported emotional burden due to the illness of their parent, 90% suffered from impaired family life, and 77% experienced burden due to the parent's symptoms. Reward (positive experience) was reported regarding the intensification of the parent-child relationship. Linear regression analysis shows predictors for highly burdened children as well as for children who are more prone to maladaptive ways of coping. Higher burden was significantly associated with the child's age, severity of illness of the parent, and specific diagnosis.

**Conclusions:**

Although some positive aspects of parental affective disorder exist, this study underlines that children primarily suffer from their parent's disorder and that this burden does not stop in adulthood. Providing professional support to adult as well as to minor children of affected individuals should become standard of care in clinical settings.

## Background

Typically, the term ‘a sample of caregivers’ refers to parents and partners but rarely to children. In studies exploring caregiver burden, children of psychiatric patients tend to comprise a small part of a heterogeneous subject group (e.g., Cleary et al. 2005; Reinares et al. 2006; Adewuya et al. 2011). Chronic psychiatric disorders, such as bipolar and depressive disorder, often end in serious problems like loss of job, poverty, or decrease of social functioning - even in times when the patient is symptom-free (Bauer et al. 2012a; Coryell et al. 1993). Of all relatives, children are the only ones being completely dependent on the psychiatric patient. There is no doubt that minor offspring of mentally ill parents experience a high level of burden (Krautgartner et al. 2007; Stelling et al. 2008) and are at a high risk for developing social, emotional, and behavioral disorders (Duffy et al. 2014; Maoz et al. 2014). Especially if they are the only caregivers of the patient, children suffer most from those psychosocial difficulties. It is known that caregivers of affected individuals show high levels of psychopathology and burden (Adewuya et al. 2011) and that those who are unable to cope effectively report the most burdensome experiences (Chakrabarti and Gill 2002). Moreover, caregiver burden continues in times of remission (Reinares et al. 2006) and is associated with patient dysfunction, duration of illness (Chakrabarti et al. 1992; Mitsonis et al. 2012), and symptom severity (Hjäthag et al. 2010).

To date, no study has been conducted that describes the burden adult children experience as a consequence of parental psychiatric disorder.

Furthermore, as far as to the last decade, the situation of caregivers was explored only as burdensome. Paradigm shift from one-sided analysis of caregiver burden to a more differentiated view of the situation of the caregivers is new (Bauer et al. 2012b). Little attention has been paid to the positive aspects and rewards of caregiving (Schwartz and Gidron 2002; Veltman et al. 2002). This one-sided focus of research does not justify the complexity of the numerous changes that result from caring for someone with a mental illness. The few studies of rewards of caregiving constrain a multitude of profit, most appreciation by the patient and others for the caregiving and satisfaction about providing care and an increase of cohesion and closer relationships with the patient and within the family (Bauer et al. 2012b, 2013; Schwartz and Gidron 2002). Reflecting reward of caregiving one's parent and developing individual coping strategies in handling mental disorder of a family member are often closely connected.

Therefore, the aim of this study was to investigate the situation - burden, reward, and ways of coping - of adult offspring of parents with bipolar disorder (OBD) and of adult offspring of parents with depressive disorder (ODD).

## Methods

### Subjects

Patients with either bipolar disorder or depressive disorder (ICD-10 F31.x and F32.x/F33.x) and who had at least one adult child were recruited consecutively 6 months along to participate in the study during their psychiatric hospitalization. Patients and adult children were informed about the study and were offered to participate voluntarily. The study protocol was approved by the ethics committee of the Medical Faculty of the University of Regensburg, and all participants gave their written informed consent to participate. One hundred twenty-four patients were contacted: 6 did not want any contact with our interviewer, 40 stated not to have children, 15 patients only had minor children, and 63 had adult children who could be included in our study. Of these 63 patients who were asked for permission to contact their adult child/children, 33 (52.4%) agreed and gave their written informed consent. Of the 33 children contacted, 30 (90.9%) agreed to participate in the study and 3 (9.1%) did not. Those 30 children came from 30 separate patients. The socio-cultural-economic background of the participants (patients as well as interviewed children) is comparable. There are no significant group comparisons in the characteristics *for the children* (ODD vs. OBD). The tested characteristics are gender, age, level of graduation, employment, marital status, common household with the patient, number of persons in the household, monthly take-home amount, hours of contact with the patient per week, distance between place of residence and clinic, legal guardianship of the patient, and taking part of a psychoeducational caregiver group. The only significant difference in the group comparisons in the characteristics *for the patients* (ODD vs. OBD) was in the psychosocial functioning (GAF). The OBD group had a significantly lower psychosocial functioning at discharge than the ODD [*Z* (*N* = 26) = −2.05; *P* < .05]. The tested characteristics are gender, age, level of graduation, employment, duration of illness, psychotherapy before admission, history of suicide attempts, clinical global impression (CGI part 1) at admission, global assessment of functioning at admission and discharge (CGI), legal guardianship, mentally ill, first-degree family members, comprehension of caregivers in patient's treatment, and situation of living of the patient after discharge.

### Instruments

Clinical records of the patients were complemented with socio-demographic variables assessed through a small questionnaire answered by all patients.

The assessment of the adult children involved a self-generated interview using a semi-structured schedule assessing both objective and subjective burden of care. Furthermore, the schedule included one question regarding reward: ‘Did you experience any positive changes in your life due to your parent's illness?’ All interviews were done by one rater (psychologist), trained by experienced psychologists in different workshops in psychological interviewing. The interviews were audio-taped and transcribed. In addition, the Freiburg Questionnaire of Coping with Disease (Freiburger Fragebogen zur Krankheitsverarbeitung, FKV, Muthny 1989) was used to assess illness adjustment. Using this questionnaire, five coping strategies can be distinguished: (i) ‘Depressive coping’ (i.e., ‘accusing himself’), (ii) ‘Active problem-orientated coping’ (i.e., ‘seeking information about illness and therapy’), (iii) ‘Distraction and building self-esteem’ (i.e., ‘showing feelings of one's own to other people’), (iv) ‘Religiousness and searching for meaning’ (i.e., ‘trying to find a sense for the illness’), and (v) ‘Trivialization and wishful thinking’ (i.e., ‘downplay the importance and relevance of the illness’). Furthermore, socio-demographic data of all children were collected.

### Analyses

The interview transcripts were analyzed using summarizing qualitative content analysis (Mayring 2000; Silverman 2001). An inter-rater reliability of *κ* = .93 was obtained. For further evaluation, a sum score, based on the number of negative/positive results reported, was calculated, which assessed all items equally.

To compare this sum score and to assess potential statistical differences between the offspring of patients with bipolar disorder (OBD) and the offspring of patients with depressive disorder (ODD), *t*-tests as well as Fisher's exact tests were used.

Linear regression analyses built by backward elimination and model refinement on the basis of the adjusted *R*^2^ were carried out to identify the variables which explained best the variance of burden and coping strategies. All clinical variables (inclusive diagnosis) regardless of the group differences were included as independent variables. The three dependent variables that were used were the sum score of reported burden, and the coping strategies ‘Depressive coping’ and ‘Trivialization and wishful thinking’, which were seen as maladaptive coping strategies.

Statistical significance was set at *α* ≤ .05. Statistical analysis was performed with the Statistical Package for Social Sciences (SPSS) software, version 16.0.

## Results

### Sample description

The patient sample consisted of 19 female (63.3%) and 11 male (36.7%) patients of whom 9 (30%) were 49 years or younger, 10 (33.3%) were between 50 and 59 years old, and 11 (36.7%) were older than 60 years. Fifteen (15) patients had bipolar disorder, and 15 had depressive disorder. The children sample consisted of 20 (66.7%) female and 10 male (33.3%) persons between 18 and 46 years old. Demographics for patients and their children are reported in Table 1.

**Table 1:** **Characteristics of the children and patient sample**

**Characteristics of the adult children sample**		**Total [** ***N*** ** = 30]**		**OBD [** ***n*** ** = 15]**		**ODD [** ***n*** ** = 15]**	
		**[** ***N*** **]**	**[%]**	**[** ***n*** **]**	**[%]**	**[** ***n*** **]**	**[%]**
Gender	Female	20	66.7	10	66.7	10	66.7
	Male	10	33.3	5	33.3	5	33.3
Age	18 to 20 years	6	20.0	5	33.3	1	6.7
	21 to 30 years	10	33.3	4	26.7	6	40.0
	31 to 40 years	11	36.7	4	26.7	7	46.7
	41 to 46 years	3	10.0	2	13.3	1	6.7
Age at onset of parental disorder	0 to 10 years	13	43.3	7	46.7	6	40.0
	11 to 20 years	6	20.0	3	20.0	3	20.0
	Over 21 years	11	36.7	5	33.3	6	40.0
Marital status	Single	13	43.3	7	46.7	6	40.0
	Living with partner	17	56.7	8	53.3	9	60.0
Child is the patient's custodian	Yes	2	6.7	2	13.3	-	-
	No	28	93.3	13	86.7	15	100.0
Contact with patient (per week)	Up to 2 h	13	43.3	9	60.0	4	26.7
	2 to 5 h	9	30.0	3	20.0	6	40.0
	5 to 15 h	2	6.7	-	-	2	13.3
	15 to 35 h	5	16.7	3	20.0	2	13.3
	More than 35 h	1	3.3	-	-	1	6.7
**Characteristics of the parent (patient) sample**		**Total [** ***N*** ** = 30]**		**F31 [** ***n*** ** = 15]**		**F32/33 [** ***n*** ** = 15]**	
		**[** ***N*** **]**	**[%]**	**[** ***n*** **]**	**[%]**	**[** ***n*** **]**	**[%]**
Diagnosis (ICD-10)	F31	15	50.0	15	100.0	-	-
	F32	4	13.3	-	-	4	26.7
	F33	11	36.7	-	-	11	73.3
Gender	Female	19	63.3	7	46.7	12	80.0
	Male	11	36.7	8	53.3	3	20.0
Age	41 to 49 years	9	30.0	4	26.7	5	33.3
	50 to 59 years	10	33.3	6	40.0	4	26.7
	60 to 69 years	11	36.7	5	33.3	6	40.0
Marital status	Single	5	16.7	1	6.7	4	26.7
	Living with partner	25	83.3	14	93.3	11	73.3
Number of children	1	7	23.3	5	33.3	2	13.3
	2	13	43.3	7	46.7	6	40.0
	3	7	23.3	3	20.0	4	26.7
	4	3	10.0	-	-	3	20.0
Ever attempted suicide	Yes	11	36.7	8	53.3	3	20.0
	No	19	63.3	7	46.7	12	80.0
Legal tutelage	Yes	4	13.3	4	26.7	-	-
	No	26	86.7	11	73.3	15	100.0
CGI (clinical global impression; severity of the disorder) at date of discharge	Mildly ill	7	23.3	2	13.3	5	33.3
	Moderately ill	6	20.0	4	26.7	2	13.3
	Considerably ill	6	20.0	4	26.7	2	13.3
	Severely ill	7	23.3	2	13.3	5	33.3
	No data provided	4	13.3	3	20.0	1	6.7

OBD = offspring of patients with bipolar disorder; ODD = offspring of patients with depressive.

Duration of the interviews was 13 to 71 min with an average of 28.6 min.

### Burden experienced due to the parent's disorder

The children's statements were summarized into 38 global statements and assigned to eight categories of burden: emotional burden (reported by 100%), burden due to impaired family life (90%), burden due to the patient's symptoms (76.7%), burden due to dissatisfaction with the patient's therapy/professional staff (73.3%), burden due to impaired free-time activities (63.3%), burden due to impaired functioning in school/job (46.7%), burden due to own health problems (43.3%), and burden due to problems in the child's relationship/own family (30.0%). Categories and statements are more precisely listed in Table 2.

**Table 2:** **Frequencies of categories and statements of burden as reported by the children sample**

**Categories and statements of reported burden**	**Total [** ***N*** **= 30]**		**OBD [** ***n*** **= 15]**		**ODD [** ***n*** **= 15]**		***p*** **(** ***a*** **)**
	**[** ***N*** **]**	**[%]**	**[** ***n*** **]**	**[%]**	**[** ***n*** **]**	**[%]**	
Emotional burden	30	100.0	15	100.0	15	100.0	1.0
• Anxiety and concern for the patient	22	73.3	14	93.3	8	53.3	.02*
• Fear of illness of one's own/hereditariness	20	66.7	10	66.7	10	66.7	.65
• Confusion and helplessness due to the patient's behavior	15	50.0	8	53.3	7	46.7	.50
• Fear of what the future might bring	15	50.0	10	66.7	5	33.3	.07
Burden due to impaired family life	27	90.0	15	100.0	12	80.0	.11
• Lack of emotional support	16	53.3	9	60.0	7	46.7	.36
• Lack of harmony/divorce	16	53.3	4	26.7	3	20.0	.50
Burden due to the patient's symptoms	23	76.7	12	80.0	11	73.3	.50
• Patient's lack of insight into the disorder	16	53.3	9	60.0	7	46.7	.36
Burden due to dissatisfaction with the patient's therapy/professional staff	22	73.3	8	53.3	14	93.3	.02*
• Lack of dedication of professional staff	15	50.0	6	40.0	9	60.0	.23
• Lack of professional support/involvement in therapy	15	50.0	7	46.7	8	53.3	.50
Burden due to impaired free-time activities	19	63.3	9	60.0	10	66.7	.50
Burden due to impaired functioning in school/job	14	46.7	10	66.7	4	26.7	.03*

Statements only listed if reported by ≥50% of all children. OBD, offspring of patients with bipolar disorder; ODD, offspring of patients with depressive disorder.

*p(a)<=0.05.

There was no significant difference between the sum score of the OBD and the ODD [*t* (*N* = 30) = −1.55; *p* = .97]. However, OBD reported significantly more burden due to impaired functioning in school/job (66.7% vs. 26.7%; *p* = .03), whereas ODD reported significantly more burden due to dissatisfaction with the patient's therapy/professional staff (93.3% vs. 53.3%; *p* = .02).

### Factors predicting the experience of burden

Adult children reported more burdensome aspects when they were either younger than 11 or older than 20 years old at the onset of their parent's disorder (*β* = −.513; *p* = .00), when severity of parent's illness was high at date of discharge (*β* = .337; *p* = .04), and when the parent was diagnosed with bipolar disorder vs. depression (*β* = 338; *p* = .04). The variables explained 47% of the total variance (*R*^2^ = 0.468; *n* = 26).

### Reward experienced due to the parent's disorder

Despite the fact that the schedule included only one question regarding reward, six categories regarding reward could be found: reward due to feeling closer to the healthy parent (reported by 46.7%; sum score OBD:ODD = 10:4), reward due to the family growing closer as a whole (23.3%; 3:4), reward due to feeling closer to the ill parent (23.3%; 2:5), reward due to the development of personality traits considered positive (13.3%; 2:2), reward due to feeling closer to the sibling(s) (6.7%; 0:2), and reward due to feeling closer to their partner/husband/wife (3.3%; 1:0).

There was no significant difference between the sum score of the OBB and the ODD [*t* (*N* = 30) = .00; *p =* .32], although OBD reported significantly more reward due to feeling closer to the healthy parent (66.7% vs. 26.7%; *p* = .03).

### Ways of coping

OBD and ODD of this sample used a wide range of coping strategies, none of which was predominant. Nevertheless, OBD used significantly more ‘Depressive coping’ (*p* = .01) and also more ‘Distraction and building self-esteem’ (*p* = .05) than ODD.

### Factors predicting maladaptive coping in adult children

Five factors were found to predict ‘Depressive coping’: ill parent being single (*β* = −.592; *p* = .06), adult child being of younger age (*β* = −.485; *p* = .01), adult child reporting more burdensome aspects (*β* = −.289; *p* = .05), adult child being in a cohabitating relationship (*β* = −.455; *p* = .01), and parent being diagnosed with bipolar disorder (*β* = −.469; *p* = .00). The variables explained 59% of the total variance (*R*^2^ = 0.586; *n* = 30).

Six factors were found to predict ‘Trivialization and wishful thinking’ best: parent (i.e., patient) not being under legal tutelage (*β* = −.768; *p* = .00), parent being single (*β* = −.302; *p* = .04), parent having attempted suicide (*β* = .299; *p* = .04), parent being diagnosed with bipolar disorder (*β* = .323; *p* = .04), adult child being 10 years or younger at the onset of the parental illness (*β* = .423; *p* = .00), and adult child being the parent's legal custodian (*β* = .558; *p* = .00). The variables explained 71% of the total variance (*R*^2^ = 0.709; *n* = 30).

## Discussion

### Specific burden of children as caregivers

Predominately, adult children of parents suffering from bipolar or depressive disorder show high levels of burden and are in urgent need of professional support. This study found aspects of burden which are special to children in the role as caregivers of psychiatric patients. While some of the children's statements have been published in previous caregiver research (Moeller-Leimkuehler 2005; Reinares and Vieta 2004; Schmid et al. 2003; Schmid et al. 2005), our results provide new details on how children experience parental affective disorder. What is more, our study compares the perceived burden of OBD and ODD. Burdens specific to children are fear of parental illness developing in oneself and/or in one's children (statement listed in category ‘emotional burden,’ reported by 66.7%; see Table 2), lack of emotional support, as well as lack of harmony/divorce (statements listed in category ‘burden due to impaired family life,’ each reported by 53.3%; see Table 2). Overall, coherence between OBD and ODD is high. However, there are two significant differences, the first of which was greater dissatisfaction with the patient's therapy/professional staff in ODD. Whereas recovery from mania can be quite fast, especially under suitable medication, this does not hold true for depression which typically requires long-term therapy: the success of which is not immediately noticeable to the patient's caregivers. This might lead to dissatisfaction with professional staff. OBD reported more burden due to impaired functioning in school/job showing that burden due to parental affective disorder continues on into adulthood. A potential reason could be that OBD worry more about both the ill parent as well as about the healthy parent since bipolar disorder is a more severe illness (e.g., low predictability of episodic course, high relapse rate, illness with two poles - depression and mania) with immense impact on family life compared to unipolar depression.

### Reward experienced by adult children as caregivers

Adult children of mentally ill parents experience an unexpectedly high amount of reward due to parental disorder. Many relatives are highly aware of positive aspects resulting from caring for a psychiatric family member (Hatfield 1997); children, in particular, are reported to be very sensitive to experiencing reward (Hinrichsen et al. 1992). Unlike other findings (Heru and Ryan 2004), OBD and ODD of this sample surprisingly did not differ in frequency of reported reward. Most positive results concern the intensification of the parent-child relationship. As adults, children of parents with psychiatric disorders are able to reflect on their childhood experiences from a more distant perspective. For most adult children of this sample, the outcome of this contemplation process seems to be not only burdensome but also a positive (learning and self-enriching) experience.

### Coping mechanisms of OBD and ODD

Children of bipolar parents are more likely to use depressive ways of coping than children of depressed parents. Previous studies focusing on caregiver coping showed that relatives of depressed and bipolar patients use many different coping styles (Adewuya et al. 2011; Chakrabarti and Gill 2002). Caregivers of bipolar patients have been shown to use problem-oriented coping more often than caregivers of depressive patients (Chakrabarti and Gill 2002; Nehra et al. 2005). In these previous studies, mostly the partner, parents, and siblings of affected individuals were interviewed. In contrast, OBD in our study do not use problem-oriented coping more often than ODD. Depressive coping thus appears to be unique to children of bipolar patients compared to other relatives. This is hardly surprising considering the fact that more than half of the bipolar patients in our sample have attempted suicide (see Table 1). On the other hand, as there is no data on the clinical state of the interviewed children, one cannot know whether this depressive coping mechanism is purely a result of the parent's conditions or whether it is somewhat contaminated by the children's own clinical course. Thus, data of occupation and income level of the children argue for a healthy sample.

Even though the adult children in our study reported some positive aspects of parental psychiatric illness, they predominately experienced high levels of burden. This study therefore proves that burden does not stop in adulthood.

### Characteristics of children who are in urgent need of professional support

High burden due to a caring for a family member with a psychiatric disorder leads to a higher risk for developing affective disorders in caregivers (Adewuya et al. 2011; Lawton et al. 1991). Children of parents with an affective disorder are more likely to develop psychiatric illnesses (Duffy et al. 2014; Nehra et al. 2005). In addition, caregiver burden influences the clinical outcome of patients with affective disorders (Perlick et al. 2001; 2004). As a consequence, supporting patients' minor and adult offspring is a crucial target of psychosocial interventions in affective disorders. Our results provide data to identify high-risk adult children who are highly emotionally burdened and thus in urgent need of professional support. According to the results of our regression analyses, characteristics of these high-risk children and their affected parent (i.e., patient) are as follows:

- Adult child is young (18 to 20 years old)
- Child was 10 years or younger at the onset of parental disorder
- Child has a cohabitating relationship
- Child is the patient's legal custodian
- Child reports many burdensome aspects
- Parent is diagnosed with bipolar disorder
- Parent has a severe disorder
- Parent is single
- Parent is not under legal tutelage
- Parent has attempted suicide

Opposite to results of previous studies (Mitsonis et al. 2012), duration of illness did not predict caregiver burden for children. In addition, contrary to other findings (Hadryś et al. 2011), children's burden did differ according to diagnostic group with bipolar disorder being associated to higher burden in children.

### Methodological considerations

Small sample size and large age range limit the degree to which these results can be generalized. Also, the interviewed children were not evaluated clinically for presence or history of any kind of psychiatric disorders. Thus, data of occupation and income level argue for a healthy sample. Most interviewed children work full time (70.0%) or part time (20%); 10% were housewives. None of them receive a pension, and the income level was relatively high. Furthermore, subjects were recruited primarily from a hospital setting and included only children who were willing to participate. These findings are, thus, limited in their applicability to other populations such as children of parents with less severe or less chronic conditions.

We have increased our knowledge about the clinical importance of supporting children of mentally ill parents. Introducing psychoeducational interventions and, as a result, reducing burden and promoting problem-oriented coping will not only diminish the children's own risk of developing psychiatric disorders but also improve the clinical outcome among patients with affective disorders (Perlick et al. 2001). Therefore, reducing burden in this high-risk group of caregivers will prevent future psychiatric illnesses. Prevention programs that are proven to have long-term positive effects on children affected by parental disorder (Beardslee et al. 2003; Siegenthaler et al. 2012) should be implemented now.

## Conclusions

Although some positive aspects of parental affective disorder exist, this study underlines that children primarily suffer from their parent's disorder in important areas of their life and that this burden does not stop in adulthood. Providing professional support to adult as well as to minor children of affected individuals should become standard of care in hospital settings.
